# Metallic 2H-Tantalum Selenide Nanomaterials as Saturable Absorber for Dual-Wavelength Q-Switched Fiber Laser

**DOI:** 10.3390/s21010239

**Published:** 2021-01-01

**Authors:** Lingling Yang, Ruwei Zhao, Duanduan Wu, Tianxiang Xu, Xiaobiao Liu, Qiuhua Nie, Shixun Dai

**Affiliations:** 1 Laboratory of Infrared Materials and Devices, The Research Institute of Advanced Technologies, Ningbo University, Ningbo 315211, China; 1811082126@nbu.edu.cn (L.Y.); zhaoruwei@nbu.edu.cn (R.Z.); wuduanduan@nbu.edu.cn (D.W.); nieqiuhua@nbu.edu.cn (Q.N.); daishixun@nbu.edu.cn (S.D.); 2 School of Sciences, Henan Agricultural University, Zhengzhou 450002, China

**Keywords:** 2H–TaSe_2_ nano-materials, metallic band structure, saturable absorber, dual-wavelength

## Abstract

A novel 2H-phase transition metal dichalcogenide (TMD)–tantalum selenide (TaSe_2_) with metallic bandgap structure is a potential photoelectric material. A band structure simulation of TaSe_2_ via ab initio method indicated its metallic property. An effective multilayered TaSe_2_ saturable absorber (SA) was fabricated using liquid-phase exfoliation and optically driven deposition. The prepared 2H–TaSe_2_ SA was successfully used for a dual-wavelength Q-switched fiber laser with the minimum pulse width of 2.95 μs and the maximum peak power of 64 W. The repetition rate of the maximum pulse energy of 89.9 kHz was at the level of 188.9 nJ. The metallic 2H–TaSe_2_ with satisfactory saturable absorbing capability is a promising candidate for pulsed laser applications.

## 1. Introduction

The increasing demand for pulsed lasers in the fields of scientific research and industrial processing has motivated researchers to explore a novel saturable absorber (SA). Materials can be divided into three categories depending on carrier concentration, namely, conductors, semiconductors, and insulators. At present, reported insulator SAs only include water [1] and alcohol [2]. The mechanism of saturable absorption is the electronic transition in vibrational energy levels of molecules. However, in-depth studies on insulator SAs are lacking. Related investigations on semiconductor SA have been conducted extensively [3,4,5]. Numerous SA materials, including SESAM, MXenes, black phosphorus, gold nanorods, and topological insulators, have been proposed to generate pulsed fiber laser. Semiconductor SAs are primarily constrained by their limited operating bandwidth because photon energies should be larger than the material bandgap, but conductor SAs can effectively solve this problem. At present, studies on conductor SAs commonly focus on metal nanoparticles [6,7], nanowires [8,9], and some special transition metal dichalcogenides (TMDs) [10].

TMDs have been regarded as graphene replacements due to their chemical structure of MX_2_ (M and X denote the transition metal and chalcogen elements, respectively) and outstanding chemical, mechanical, and optoelectronic properties [11]. Given the abundance of M and X, TMDs have a large family with different characters. The chalcogen element X determines the stability and lattice parameter of TMDs, while the transition metal M influences electronic properties. Hence, TMDs can be categorized as semimetallic, metallic, or even superconducting materials [12,13]. Semiconductive TMDs, such as MoS_2_, and WSe_2_, have been extensively investigated [4]. However, metallic TMDs have rarely been explored. As a metallic TMD, 2H–tantalum selenide (TaSe_2_) demonstrates unique magnetic, superconducting, and optical properties. Although the strain-induced ferromagnetism of monolayer TaSe_2_, charge density wave, and functionality in logic circuits and switches have been investigated [14,15,16], reports on nonlinear optical properties of metallic 2H–TaSe_2_ are limited.

The band structure and optical properties of 2H–TaSe_2_ were investigated in this study. According to density functional theory, monolayer and multilayer 2H–TaSe_2_ materials both exhibit a metallic electronic band structure. The fiber-integrated few-layered 2H–TaSe_2_ SA was fabricated using liquid-phase exfoliation and optically driven deposition method. Open-aperture Z-scan measurement examined the saturable absorption property of 2H–TaSe_2_. A dual-wavelength Q-switched fiber laser with a pulse energy and peak power of 188.9 nJ and 64 W, respectively, was demonstrated using the prepared 2H–TaSe_2_ SA. The satisfactory nonlinear optical modulating ability of 2H–TaSe_2_ proves its potential in novel optoelectronic applications.

## 2. Band Structure and Characterization of TaSe_2_-SA

**Figure 1:**
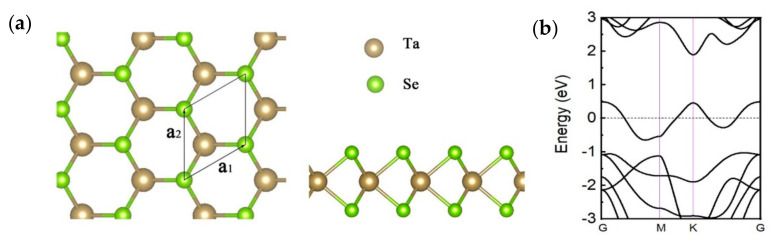
(**a**) Top (left) and side (right) view of monolayer TaSe_2_ and (**b**) band structures of monolayer TaSe_2_. Fermi level is set to 0 eV. G (0, 0, 0), M (0.5, 0, 0), and K (0.333333, 0.333333, 0) represent highly symmetric points in the reciprocal space.

**Figure 2:**
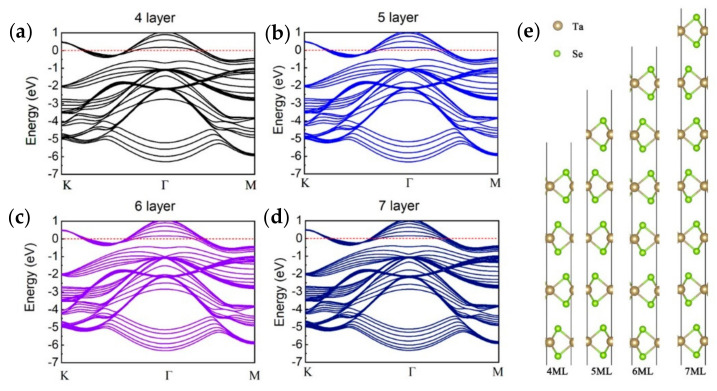
Band structures for multilayer TaSe_2_: (**a**) four-, (**b**) five-, (**c**) six-, and (**d**) seven-layer TaSe_2_; (**e**) corresponding atomic-layered structures of TaSe_2_.

The atomic-layered structure of monolayer 2H–TaSe_2_ is illustrated in Figure 1a. Similar to other TMDs, the transition metal (Ta) layer is sandwiched between two chalcogen (Se) layers. Our first-principle calculations were performed using Vienna Ab initio simulation package known as the VASP code [17,18,19]. The electronic–ion interaction is described using projector-augmented wave method [20]. Energy cutoff of plane waves was set to 520 eV. The electron-exchange correlation function was treated using generalized gradient approximation (GGA) in the form proposed by Perdew, Burke, and Ernzerhof (PBE) [21]. Both atomic positions and lattice vectors were fully optimized using conjugate gradient (CG) algorithm with an energy precision of 10–5 eV until the maximum atomic forces are smaller than 0.008 eV/Å. A vacuum region of approximately 15 Å was adapted to eliminate the interaction of two adjacent images. Brillouin zone (BZ) integration was sampled using a 17 × 17 × 1 k-mesh according to Monkhorst–Pack method [22]. The calculation above confirmed that 2H–TaSe_2_ possesses a metallic and electronic band structure, as shown in Figure 1b. Varying band structures of 2H–TaSe_2_ from 4 layers to 7 layers were further calculated using the same method, and the results are illustrated in Figure 2a–d. The corresponding atomic-layered structures are presented in Figure 2e. The calculated band structures demonstrated a small difference with the results of a previous report [23] because of the different settings in calculation parameters.

The fiber-integrated few-layered TaSe_2_ SA was fabricated using liquid-phase exfoliation and optically driven deposition method. Bulk TaSe_2_ material was grinded into powder for intensive mixing with isopropyl alcohol (IPA). The initial mixture was then sonicated in an ultrasonic bath for approximately 8 h. Notably, this process has a suitable interval to prevent dispersion overheating. Finally, the as-prepared solution was centrifuged at 1500 rpm for 15 min to separate the large agglomeration, and the upper TaSe_2_ supernatant was decanted and set aside.

Homogeneously dispersed solution was dropped on a sapphire substrate to test the characterization of TaSe_2_ nanosheets conveniently. Then, the substrate was dried under an infrared oven lamp for approximately 5 h to fabricate the TaSe_2_ sample. Then the optically driven deposition method was applied to form a fiber-integrated few-layered TaSe_2_ SA. Light with a wavelength and outpower of 980 nm and 60 mW, respectively, was directly irradiated onto the fiber. Fiber ferrule was dipped into TaSe_2_ dispersion for 15 min. After drying for 24 h at room temperature, the fiber-integrated TaSe_2_ SA was formed by connecting it with a clean one using an adaptor.

**Figure 3:**
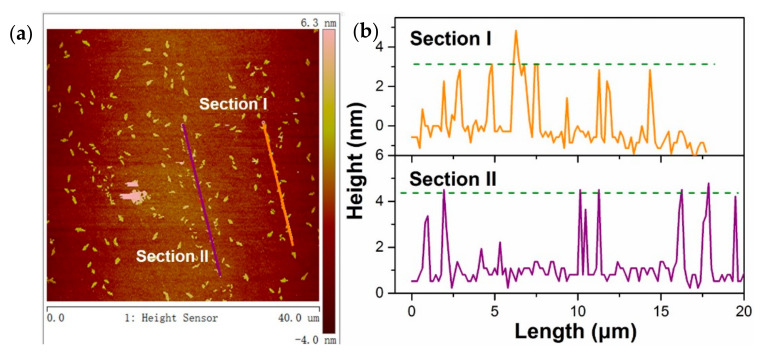
(**a**) Atomic force microscope (AFM) image of the 2H–TaSe_2_ sample in a 40 × 40 μm region and (**b**) corresponding height.

As-prepared TaSe_2_ nanosheets were observed using AFM to investigate the morphology and layer character. The image area (40 μm × 40 μm) is illustrated in Figure 3a. A homodisperse solution was successfully fabricated. The corresponding cross-section analysis is shown in Figure 3b. The green dotted line denotes the average height, which showed that the thickness of the 2H–TaSe2 sample is approximately 3–5 nm; thus, the fabricated sample is a 4–7-layered structure (single-layered TaSe_2_ shows a thickness of around 0.75 nm [24,25]).

**Figure 4:**
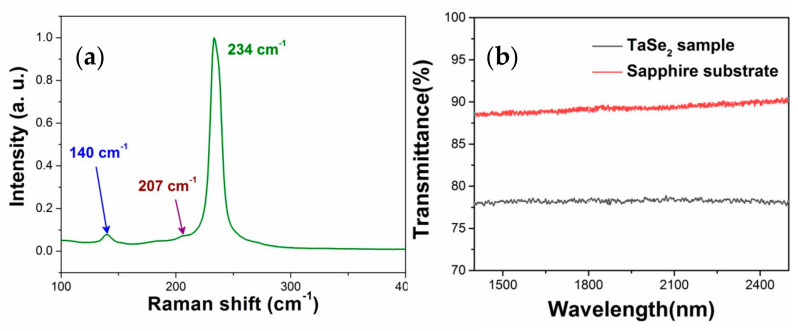
(**a**) Raman data of 2H-TaSe_2_ nanosheets. (**b**) Linear transmittance of the sample and sapphire substrate.

The phase of the TaSe_2_ sample was confirmed by investigating its Raman spectrum at room temperature using a laser source with an excitation of 532 nm (Renishaw inVia Raman microscope with the spectral resolution of 1 cm^−1^). The 2H–TaSe_2_ sample in the metallic phase demonstrates hexagonal syngony of $D_{6h}^{4}$ space group. A_1g_ and E_1g_ (E_2g_) Raman active modes represent the out-of-plane and in-plain vibrational modes, respectively. Figure 4a shows that Raman peaks located at 140, 207, and 234 cm^−1^ correspond to E_1g_, E_2g_, and A_1g_ modes, respectively. Compared with previous reports, some negligible shifts of peak position and intensity caused by the difference in layer numbers of TaSe_2_ nanosheets were observed [25,26]. Linear transmission spectra of the TaSe_2_ sample and sapphire substrate in Figure 4b were examined using a spectrophotometer (U-3500) to show the broadband absorption at infrared band of metallic nanomaterial.

**Figure 5:**
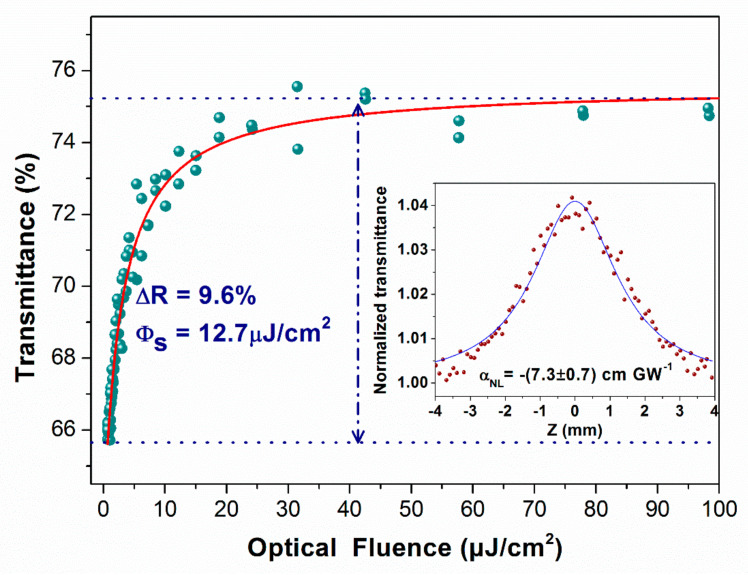
Saturable absorption curve of 2H-TaSe_2_. (Note: The inset shows the open-aperture Z-scan data).

An open-aperture Z-scan was applied to assess the nonlinear optical character of 2H–TaSe_2_. A homemade pulsed fiber amplifier (wavelength, pulse width, and repetition rate of 1560 nm, 15 ps, and 1 MHz, respectively) was used to measure the Z-scan curve as follows (inset of Figure 5) [27]:

(1) Tz=∑m=0∞−αNLI0Leffmm+11.5zz02+1m≈1−αNLI0Leff22zz02+1+αNLI0Leff233zz02+12

where *L_eff_* is the effective length, *α_NL_* is the nonlinear optical coefficient, *I*_0_ is the power intensity, *z*_0_ is the Rayleigh length, and *T* is the normalized transmittance. The nonlinear optical coefficient *α_NL_* was (7.3 ± 0.7) cm/GW, according to the fitting result, which is comparable with the findings of other mature 2D materials [28]. According to the change in beam radius of the Gauss beam, the saturable absorption curve can be extracted based on Z-scan data, as illustrated in Figure 5. The modulation depth and saturation intensity were separately calculated at 9.6% and 12.7 μJ/cm^2^, respectively, through data fitting [27]. The results showed that the metallic 2H–TaSe_2_ sample can be a SA candidate for generating pulse lasers.

## 3. Experimental Setup

**Figure 6:**
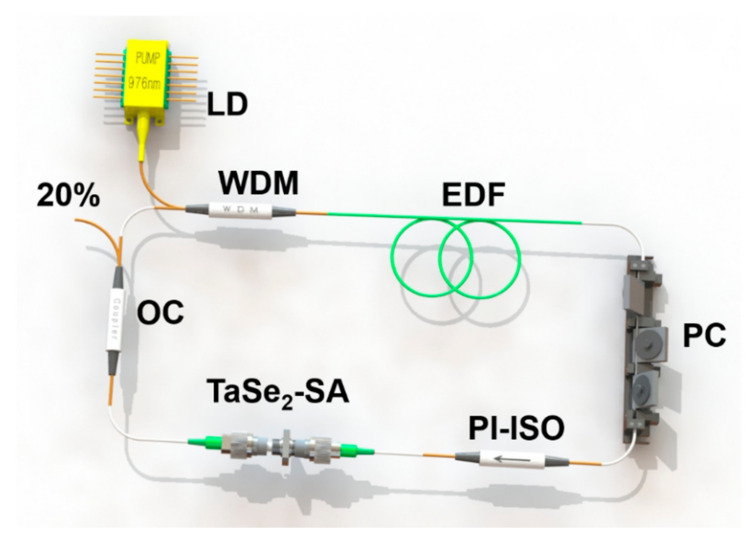
Experimental setup of the Q-switched fiber laser.

The Q-switched all-fiber laser cavity based on TaSe_2_ SA is illustrated in Figure 6. The laser cavity length, including the 0.5 m-long and highly erbium-doped (EDF, LIEKKI: Er110-4-125) and else-tailed (single-mode fiber, SMF) fibers, was approximately 7.5 m. As a commercial 980 nm laser diode (LD) with a maximum power of 550 mW, the pump source is used via 980/1550 nm wavelength division multiplexing (WDM) to pump EDF with a dispersion parameter of −12 ps/nm/km. A polarization controller (PC) and a polarization-independent isolator (PI-ISO) were applied to optimize cavity birefringence and ensure the unidirectional operation of the laser cavity, respectively. Moreover, 20% of the energy of an optical coupler (OC) is used to output laser signal. Given that the standard SMF shows a dispersion parameter of 18 ps/nm/km, the net cavity dispersion is calculated at −0.153 ps^2^.

## 4. Results and Discussion

**Figure 7:**
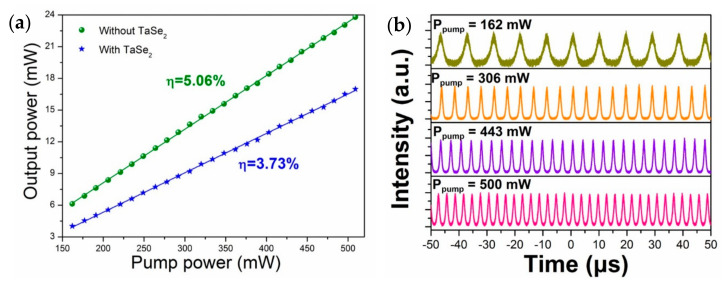
(**a**) Output power versus pump power with TaSe_2_ (green dots) and without TaSe_2_ (blue stars). (**b**) Stable pulse trains with different pump power values.

A clean ferrule without deposition was first inserted into the fiber cavity to prove the optical modulation capability of the TaSe_2_ nanomaterial. As a result, Q-switch was absent, except continuous wave (CW), regardless of the pump power change or PC tuning. The output power was measured with a power meter (Thorlabs, PM100D), and its change versus pump power without TaSe_2_ is illustrated in Figure 7a (green dots). The slope efficiency was 5.06% with the maximum output power of 23.8 mW under a pump power of 500 mW. The maximum output power decreased to 16.9 mW with a slope efficiency of 3.73% when the prepared TaSe_2_ SA was inserted into the cavity due to the insertion loss induced by TaSe_2_ SA. The results indicated the successful deposition of TaSe_2_ materials onto the fiber ferrule.

**Figure 8:**
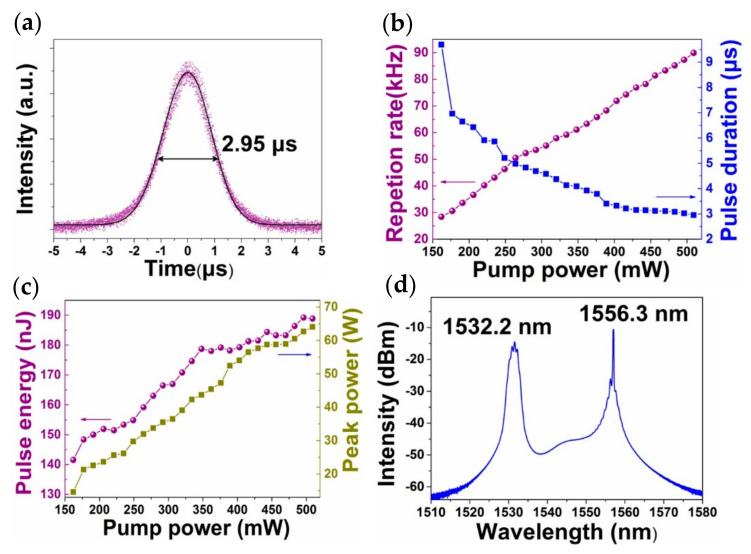
Laser performance: (**a**) Minimum pulse profile with a Gaussian-fitting standard deviation of 7.36 × 10^−4^. (**b**) Pulse width and repetition rate versus pump power. (**c**) Pulse energy and peak power versus pump power. (**d**) Output spectrum with a pump power of 500 mW.

Stable and typical Q-switched pulses were observed when the pump power was increased to 160 mW. Figure 7b shows the output pulse trains with different pump power values. The evidently stable output pulses with the maximum pump power (500 mW) indicated the high thermal damage threshold of the metallic TaSe_2_ material. When the pump power increased from 160 to 500 mW, the rate repetition increased from 28.4 to 89.9 kHz but the pulse width reduced from 9.69 to 2.95 μs. The minimum pulse with a Gauss-pulse profile assumed and pulse width (repetition rate) that depend on the pump power are depicted in Figure 8a,b, respectively. The change mechanism can be explained by the population inversion. The increase in electron accumulation on the upper energy level with increasing pump power shortens the rising and following time of big pulse formed by the sudden release of stored energy released suddenly. As a result, the pulse width reduced and the rate repetition increased with the increment of pump power. The pulse energy and peak power at different pump power values can be easily calculated, as shown in Figure 8c. The maximum pulse energy and peak power were 188.9 nJ and 64 W, respectively, at an average output power of 16.9 mW.

The output spectrum is illustrated in Figure 8d. A dual wavelength with spectral separation of 24.1 nm was detected. Methods of multiwavelength fiber laser generation, such as subzero treatment [29], Mach–Zehnder interferometry element [30,31,32], and highly nonlinear materials [33,34], have been extensively investigated. Two oscillation wavelength peaks were located at 1532.2 and 1556.3 nm while the oscillation wavelength of the CW state demonstrated only one peak at 1564.6 nm in this study. Therefore, the generation of dual-wavelength Q-switched lasers was caused by the high nonlinearity of the nanomaterial [33,34].

**Figure 9:**
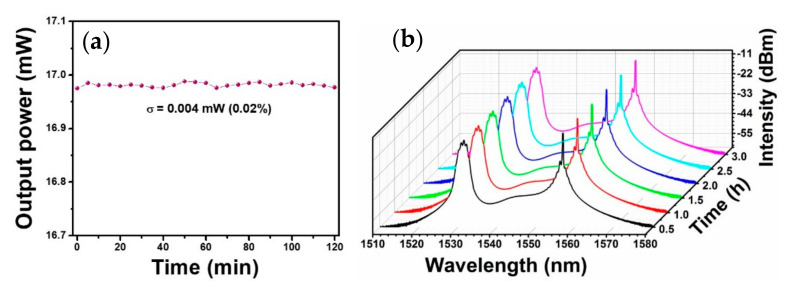
Long-term stability of (**a**) output power with the standard deviation of 0.004 mW and (**b**) output spectra at a pump power of 500 mW.

Finally, we explored a 2 h output power at a pump power of 500 mW (Figure 9a). The standard deviation of measured output power is 0.004 mW and the corresponding RMS (standard deviation/average output power) is 0.02%. Output spectra versus time at a pump power of 500 mW was also monitored (Figure 9b). The absence of evident vibrations indicated the excellent environmental stability of the Q-switched EDF lasers. Besides, Table 1 summarized the performance of Q-switched fiber laser using common semiconductor TMDs (including ternary TMD) materials as saturable absorber. By contrast, the 2H-TaSe_2_ exhibited a comparable optical modulation ability. In view of the metallic band-structure, it could be deduced the 2H-TaSe_2_ would be an except full-wave-band optical modulator.

## 5. Conclusions

A novel 2H–TaSe_2_ with a metallic electronic band structure was used as a SA to generate Q-switched fiber laser. The nonlinear optical coefficient, modulation depth, and saturation intensity were (7.3 ± 0.7) cm/GW, 9.6%, and 12.7 μJ/cm^2^, respectively, via open-aperture Z-scan measurement. We demonstrated a dual-wavelength Q-switched fiber laser with a minimum pulse width of 2.95 μs based on the prepared 2H–TaSe_2_ SA. The maximum pulse energy of 188.9 nJ and maximum peak power of 64 W were calculated at the maximum repetition rate of 89.9 kHz. The excellent nonlinear optical modulating ability of 2H–TaSe_2_ verifies its potentiality in novel optoelectronic applications as a metallic TMD material.

## Figures and Tables

**Table 1:** Typical Q-switched EDF lasers using common transition metal dichalcogenides (TMDs) as saturable absorbers (SAs).

Materials	Modulation Depth	Saturation Intensity	Minimum Pulse Width (μs)	Pulse Energy (nJ)	Ref.
MoS_2_	29%	4.53 MW/cm^2^	6	150	[35]
WS_2_	7.7%	342.6 MW/cm^2^	0.1549	68.5	[36]
MoSe_2_	6.73%	132.5 MW/cm^2^	4.04	365.9	[37]
WSe_2_	7.17%	7 MW/cm^2^	1	29	[38]
MoWSe_2_	19.7%	18.9 μJ/cm^2^	1.53	151.4	[27]
2H-TaSe_2_	9.6%	12.7 μJ/cm^2^	2.95	188.9	This work

## Data Availability

Data sharing is not applicable to this article.
